# Performance Evaluation of Ultrasound Images Using Non-Local Means Algorithm with Adaptive Isotropic Search Window for Improved Detection of Salivary Gland Diseases: A Pilot Study

**DOI:** 10.3390/diagnostics14131433

**Published:** 2024-07-04

**Authors:** Ji-Youn Kim

**Affiliations:** Department of Dental Hygiene, Gachon University, 191, Hambakmoero, Yeonsu-gu, Incheon 21936, Republic of Korea; hoho6434@gachon.ac.kr; Tel.: +82-32-820-4376

**Keywords:** ultrasound image, noise reduction method, adaptive non-local means algorithm, quantitative evaluation of image quality

## Abstract

Speckle noise in ultrasound images (UIs) significantly reduces the accuracy of disease diagnosis. The aim of this study was to quantitatively evaluate its feasibility in salivary gland ultrasound imaging by modeling the adaptive non-local means (NLM) algorithm. UIs were obtained using an open-source device provided by SonoSkills and FUJIFILM Healthcare Europe. The adaptive NLM algorithm automates optimization by modeling the isotropic search window, eliminating the need for manual configuration in conventional NLM methods. The coefficient of variation (COV), contrast-to-noise ratio (CNR), and edge rise distance (ERD) were used as quantitative evaluation parameters. UIs of the salivary glands revealed evident visualization of the internal echo shape of the malignant tumor and calcification line using the adaptive NLM algorithm. Improved COV and CNR results (approximately 4.62 and 2.15 times, respectively) compared with noisy images were achieved. Additionally, when the adaptive NLM algorithm was applied to the UIs of patients with salivary gland sialolithiasis, the noisy images and ERD values were calculated almost similarly. In conclusion, this study demonstrated the applicability of the adaptive NLM algorithm in optimizing search window parameters for salivary gland UIs.

## 1. Introduction

Salivary glands produce and secrete saliva and are classified into the following two types: three pairs of major salivary glands and numerous minor salivary glands [1]. The major salivary glands are the parotid, submandibular, and sublingual glands, which secrete saliva for the digestion of carbohydrates [2]. Abnormal function of salivary glands and decreased saliva output lead to dry mouth and loss of self-cleaning action, simultaneously causing rampant caries and viral infections [3]. Acute inflammation, benign and malignant tumors, and sialolithiasis lesions are mainly observed in the salivary glands, and the location of these lesions varies depending on their characteristics [4,5,6,7].

Various medical imaging technologies have been used to diagnose salivary gland lesions. Fluoroscopy and computed tomography are the most commonly used radiological examinations to diagnose salivary gland lesions, and nuclear medicine imaging using radioisotopes is used depending on the patient’s situation [8,9,10,11]. However, these imaging technologies have increased radiation exposure to patients because they use X-rays or gamma rays [12]. An imaging technology that uses ultrasound to diagnose salivary gland lesions has received attention as it overcomes these shortcomings [13,14,15].

Ultrasound imaging technology is well known in the field of diagnostic medicine as a method for noninvasively observing lesions. To acquire ultrasound images (UIs), a probe or transducer is essential, and an appropriate frequency must be set based on the area to be observed. In general, using high-frequency ultrasound enables observing organs close to the skin and improving axial resolution. Salivary glands are classified as superficial organs, and scans usually include a high-frequency linear probe to diagnose lesions. When observing benign and malignant tumors and sialolithiasis of the salivary glands using UIs, the exact diameter of each lesion displayed on the screen should be measured. However, in imaging based on high-frequency ultrasound, the influence of speckle noise increases, inevitably causing blurred lesion boundaries. Speckle noise is a type of noise that occurs when ultrasound waves interfere with each other and is a major factor in the deterioration of image quality.

Technologies to reduce speckle noise are required to accurately distinguish and diagnose lesions using UIs, and software-based methods are widely used. Both hardware and software methods are being actively studied to remove speckle and electrical signal-induced Gaussian noise from ultrasound waves. Hardware-based noise rejection methods use high-quality transducers. Research on the development of high-frequency ultrasound transducers, including piezoelectric thin films and single-crystal materials [16], and high- and multi-frequency transducers is underway [17]. High-quality transducers improve image quality; however, the higher frequencies provide better resolution but lower penetration, and lower-frequency transducers provide deeper penetration but lower resolution. Beamforming is another effective method for noise removal. In particular, adaptive beamforming can be used to optimize the signal-to-noise ratio for depth [18]. Methods with acoustic matching layers and noise rejection with damping materials use materials with acoustic properties that match human tissue to reduce reflections and noise or apply damping materials to the transducer to minimize mechanical vibration and the resulting noise [19,20,21]. Hardware-based noise rejection is effective because it removes physically generated distortions. However, it may require additional devices and materials or display performance differences, depending on the system environment.

Software-based noise reduction methods do not require additional work because they perform distortion correction based on the information obtained. The Gaussian and median filter methods are traditional approaches for removing noise from ultrasound and medical images [22]. Although these methods are effective at removing noise components, they are limited in their ability to remove noise while preserving the edges. To overcome this problem, a noise reduction method based on different approaches was introduced. In noise reduction based on the wavelet transform, efforts have been made to minimize edge information loss by denoising each sub-band frequency [23]. An anisotropic diffusion-based noise reduction method was also introduced to match the speckle noise characteristics [24]. It was specifically designed to reduce speckle noise in UIs while preserving the edges and other important features. However, its performance is highly dependent on the parameters, such as the diffusion coefficient and the number of iterations. Incorrect parameter choice can result in insufficient noise reduction or excessive smoothing, thereby blurring important image details. The total variation (TV)-based noise reduction method is effective in reducing noise while preserving significant edges. However, TV regularization sometimes causes staircase artifacts because it favors partially constant solutions [25]. Further, the non-local means (NLM) approach is modeled based on the weighting principle by averaging similar patches across the image, preserving fine details and textures [26]. Kim et al. used a non-local means noise reduction method to improve UI quality for the accurate diagnosis of thyroid nodules. An ATS-539 multipurpose phantom was used to scan the dynamic range and grayscale measurement areas that were most relevant to the noise level. The scans were performed using a 3.5 MHz frequency probe, and the algorithm was modeled to achieve precise quality control and accurate diagnosis. Furthermore, the proposed NLM noise reduction algorithm could improve the UI accuracy for abdominal diseases and the thyroid region [27].

The traditional NLM method uses a fixed-size search window on every pixel to reduce the noise in an image, and its performance degrades as the noise variance increases. When a pixel is located in a smooth area, the search window must be large enough to estimate the original pixel value. In contrast, when a pixel is located in a non-smooth area, the search window must be small enough to effectively preserve the edges and textures. Verma et al. proposed an adaptive NLM method with an adaptive isotropic search window size based on the gray-level difference (GLD) of each pixel [28]. Based on normal optical images, they demonstrated that the adaptive NLM has the advantage of retaining meaningful edge information by adapting the window size to the context of each pixel.

In this study, we investigated an adaptive NLM method to improve image quality in salivary gland UIs. The objective of this study was to perform a quantitative evaluation of image quality using the adaptive NLM method, compare it with that of conventional TV and NLM methods, and verify the viability of the algorithm. The proposed method has the following two characteristics:

(1) Adaptive noise reduction can be performed to account for inter-regional noise characteristics and avoid unnecessary sharpness reduction in UIs.
(2) Comparing with existing algorithms, quantitatively evaluate noise reduction level and edge preservation ability in UIs.

In this study, the proposed algorithm was investigated, and its feasibility was evaluated experimentally. In the following sections, the proposed method, evaluation factors, and experimental results are briefly described and discussed.

## 2. Materials and Methods

### 2.1. Ultrasound Image

The UI data used in this study were obtained from a free ultrasound library provided by SonoSkills and FUJIFILM Healthcare Europe (https://www.ultrasoundcases.info/cases/head-and-neck/salivary-glands, accessed on 1 March 2024). This is the world’s largest ultrasound case library, featuring over 7000 cases, more than 100,000 ultrasound images, and 2500 clips. It was initiated in 2004 by Dr. Taco Geertsma, with support from Fujifilm Healthcare Europe. The dataset includes numerous general ultrasound cases, accumulated during his many years at Gelderse Vallei Hospital in Ede, the Netherlands. These cases have been collected over the years by the hospital’s radiologists and ultrasound technicians. The website was developed in close collaboration with Fujifilm Healthcare Europe. The salivary gland area was selected from the provided database, and images of patients with malignant tumors and sialolithiasis that were appropriate for UI denoising were used.

### 2.2. Process of Adaptive NLM Method in Ultrasound Image

**Figure 1:**
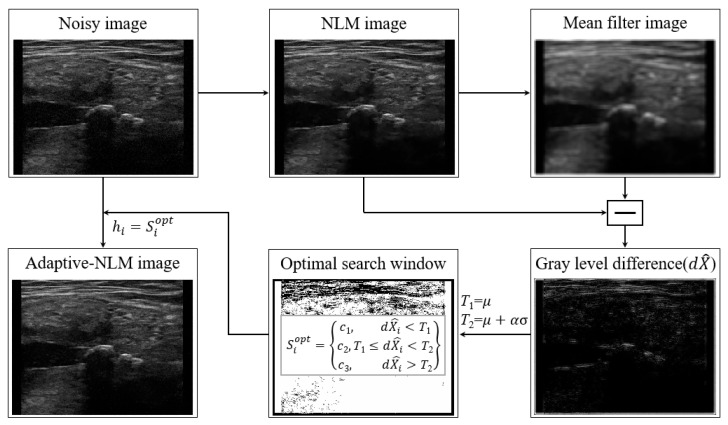
Process of the adaptive non-local means (NLM) method in UI. The optimal search window is calculated using the gray-level difference (GLD) approach.

Figure 1 shows the simple process of the adaptive NLM method for an UI. Briefly, noisy images were acquired using an ultrasound imaging system. First, simple noise reduction was performed using basic NLM methods. The conventional NLM approach for noise reduction is based on Equation (1) [27]:

(1) $$I\left(i\right)=\sum_{j\in N_{i}}w_{ij}I\left(j\right),\sum_{j}w\left(i,j\right)=1,\;0\leq w\left(i,j\right)\leq 1,$$

where $I\left(i\right)$ is the intensity of pixel i, $N_{i}$ is the search window value at pixel i, $w_{ij}$ is the weight value between the i and j pixels, and $I\left(j\right)$ is the intensity of pixel j. $w_{ij}$ represents the weight and the similarity between pixels i and j. $w_{ij}$ is defined as follows:

(2) $$w_{ij}=\frac{1}{Z\left(i\right)}{exp}^{-\frac{{\left\|p\left(i\right)-p\left(j\right)\right\|}_{2}^{2}}{h^{2}}},\;Z\left(i\right)=\sum_{j}{exp}^{-\frac{{\left\|p\left(i\right)-p\left(j\right)\right\|}_{2}^{2}}{h^{2}}},$$

where $p\left(i\right)$ and $p\left(j\right)$ represent the areas within the search window and both windows are equal in size. $Z\left(i\right)$ is the normalization constant for the similarity between two square patches $p\left(i\right)$ and $p\left(j\right)$ centered at pixels i and j, with h serving as the filtering parameter. The Gaussian kernel size was set to 11, the distance between $p\left(i\right)$ and $p\left(j\right)$ was 3, and h was 3. The weight distribution was calculated to increase around pixels with similar patterns and was adjusted based on the Euclidean distance.

After applying the mean filter to the results of the basic NLM to obtain the smoothing results, the absolute error value of each pixel was calculated to obtain the GLD ($d\hat{X}$) [28]. The size of the mean filter was 3 × 3 empirically. The first threshold value was the average value (μ) of the GLD, and the second threshold value was calculated by adding the standard deviation (σ) of the GLD multiplied by the weight (α) to the average value of the GLD. Here, the α value of the study was 0.5, as a rule of thumb. The optimal search window ($S_{i}^{opt}$) was calculated as follows:

(3) $$S_{i}^{opt}=\left\{\begin{array}{c} c_{1},\;d\hat{X_{i}}<T_{1}\\ c_{2},\;T_{1}\leq d\hat{X_{i}}<T_{2}\\ c_{3},\;d\hat{X_{i}}>T_{2} \end{array}\right\},$$

where the value $c_{1}$ is assigned if the value of $d\hat{X_{i}}$ is less than $T_{1}$, $c_{2}$ is assigned if the value of $d\hat{X_{i}}$ is between $T_{1}$ and $T_{2}$, and $c_{3}$ is assigned if the value of $d\hat{X_{i}}$ is greater than $T_{2}$ at pixel *i*. Here, the values of $c_{1}$, $c_{2}$, and $c_{3}$ were multiplied by 0.7, 0.9, and 1.1 times the h values used in the conventional NLM, respectively. These constants were not fixed and could be changed by the user depending on the characteristics of the image used and the amount of noise. Finally, the optimal search window obtained using the conventional NLM algorithm on each pixel was applied to the h value to obtain optimal noise reduction in the noisy image.

### 2.3. Quantitative Evaluation Parameters of Ultrasound Images

The coefficient of variation (COV) and contrast-to-noise ratio (CNR) were used to quantitatively evaluate the noise level of the acquired salivary gland UIs. The lower the COV and the higher the CNR, the more efficient the noise reduction in the UI. The equations for the COV and CNR evaluation parameters are as follows [29]:

(4) $$COV=\frac{\sigma_{target}}{\mu_{target}}$$

(5) $$CNR=\frac{\left|\mu_{target}-\mu_{background}\right|}{\sqrt{\sigma_{target}^{2}+\sigma_{background}^{2}}}$$

where $\mu_{target}$ and $\sigma_{target}$ are the expected mean value and standard deviation for the established target region of interest (ROI), respectively; and $\mu_{background}$ and $\sigma_{background}$ are the expected mean value and standard deviation for the established background ROI, respectively.

**Figure 2:**
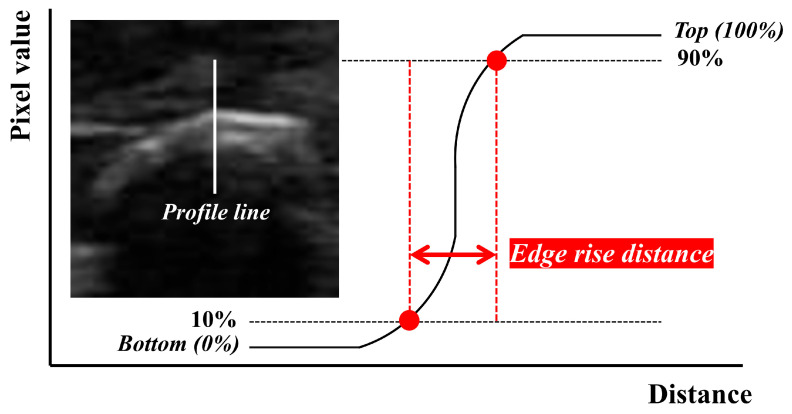
Schematic diagram for measuring edge rise distance (ERD) in the ultrasound sample image (sialolithiasis area in the submandibular gland). ERD is the distance required to rise from 10% to 90%, measured in the intensity profile.

The edge rise distance (ERD) evaluation parameter was used to evaluate the edge preservation ability of the acquired UIs. ERD refers to the distance or value required for the edge response in an image to increase from 10% to 90%, and Figure 2 shows an example of the measurement method [30].

**Figure 3:**
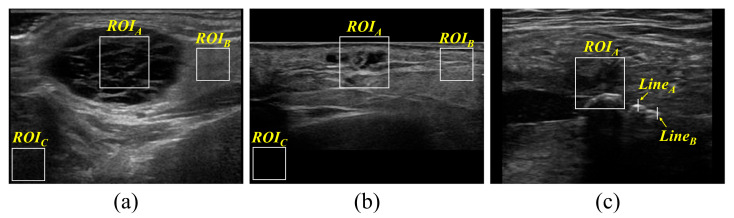
Sample UIs showing the ROI set to measure noise level and edge preservation. The ROI_A_ area marked in all the images was set for visual assessment. (**a**) ROI_B_ and (**b**) ROI_C_ were set to calculate the coefficient of variation (COV) and contrast-to-noise ratio (CNR) (COV: ROI_B_; CNR: ROI_B_ and ROI_C_). (**c**) Edge rise distance was calculated using Line_A_ and Line_B_ sets.

The ROIs for evaluating the visual assessment, noise level, and edge preservation of the acquired UIs are shown in Figure 3.

## 3. Results and Discussion

**Figure 4:**
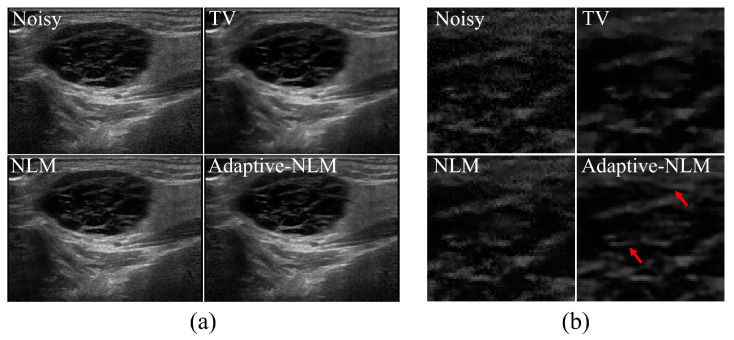
Results of applying noise reduction methods to the UIs of malignant tumors in the parotid area of the salivary glands. (**a**) Noisy image from applying conventional TV, the NLM algorithm, and the proposed adaptive NLM algorithm, and (**b**) UI resulting from enlarging the ROI_A_ area in Figure 3a. When using the adaptive NLM algorithm in the red arrow area, a clear edge line is visible in the internal echo part of the malignant tumor area.

**Figure 5:**
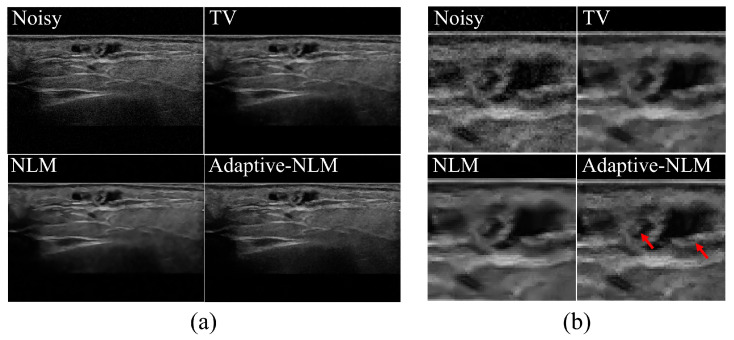
Results of applying the noise reduction methods to the UI after 1 year in the patient with parotid malignant tumors shown in Figure 4. (**a**) Noisy image from applying conventional TV, the NLM algorithm, and the proposed adaptive NLM algorithm, and (**b**) UI resulting from enlarging the ROI_A_ area in Figure 3b. The red arrow indicates that the edge can be clearly identified, which is one of the ultrasound imaging findings of malignant tumors.

Figure 4 shows the resulting images based on the noise reduction method in the UI of a patient with a malignant tumor in the parotid gland. Figure 4a shows the UIs obtained by applying the noise reduction method, including the noisy image and the proposed adaptive NLM algorithm, and (b) shows the enlarged image obtained using the ROI_A_ area shown in Figure 3a. Figure 5 shows the UI results of the malignant tumor area scanned one year later in the same patient shown in Figure 4. Figure 5a shows the entire UI obtained using noise reduction methods, and (b) shows the results of enlarging the ROI_A_ area shown in Figure 3b.

Clinical ultrasound imaging technology can be used effectively for diagnosing diseases of the salivary gland, a superficial organ. However, while some studies have found that clinical ultrasound imaging can distinguish between benign and malignant parotid tumors with high accuracy, others have provided conflicting conclusions [31,32,33]. Therefore, it is very important to distinguish the type of salivary gland tumor by maximizing the quality of UIs. According to Wu et al., irregular and heterogeneous forms of parotid tumors were confirmed in approximately 23% and 81% of patients, respectively [33]. Improving the quality of the UIs of these complex structures is essential for distinguishing between malignant and benign tumors.

Ultrasonographic findings of the parotid gland typically show echoes similar to those of the thyroid gland. In contrast, the ultrasound findings of malignant tumors of the parotid gland are nonspecific, have relatively clear boundaries, and may be accompanied by posterior acoustic enhancement. Noise and unclear boundaries have a significant impact on distinguishing between benign and malignant tumors of the salivary glands using ultrasound imaging. When the adaptive NLM algorithm proposed in Figure 4 and Figure 5 was applied to the salivary gland UIs, it was confirmed that the visually effective noise level was reduced. As shown by the red arrow in Figure 4b, it was confirmed that the edge of the gland appears more clearly in the internal ultrasound echo pattern of the salivary gland malignant tumor. In addition, in the area indicated by the red arrow in Figure 5b, the proposed algorithm can clearly distinguish the overall outline of the tumor in the UI one year after the malignant tumor of the salivary gland was observed.

Ultrasound examination of salivary gland diseases requires high-quality images due to the nature of the superficial organs, and Doppler examination is necessary because of the number of important vascular structures. During Doppler examination of malignant tumors, the resistance index increases because of the increased resistance of blood vessels, and fewer blood flow signals are detected. The degree of decreased blood flow on color Doppler examination provides useful clinical information when patients with symptomatic salivary gland disease are monitored continuously, even after their symptoms have improved [34,35]. Accurate UI quality is crucial to distinguishing between malignant and benign salivary gland tumors during a Doppler examination. The proposed adaptive NLM algorithm will have great application potential in high-performance Doppler examination for identifying tumor shape with efficient noise reduction based on appropriate edge preservation ability.

**Figure 6:**
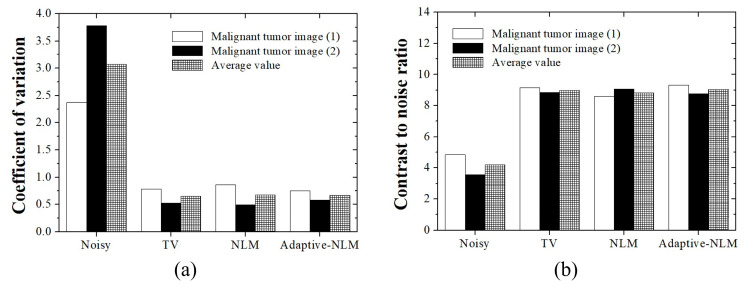
Graph showing (**a**) coefficient of variation (COV) and (**b**) contrast-to-noise ratio (CNR) values calculated from the UI of the salivary gland malignant tumor using each noise reduction algorithm. Excellent COV and CNR values were achieved using the proposed adaptive NLM algorithm.

Figure 6a,b show the COV and CNR results, respectively, as a function of the noise reduction method for the UIs of the salivary gland malignant tumor. The COV and CNR results are expressed as the average of the values measured in the ROI_B_ and ROI_C_ areas in Figure 3a,b. The average COV values obtained using the UI of the salivary gland malignant tumor were 3.08, 0.65, 0.68, and 0.67 for the noisy image, TV, NLM, and the proposed adaptive NLM algorithms, respectively. Therefore, the best COV value was derived from the UI of the malignant tumor captured using the proposed adaptive NLM algorithm, and this value was an approximately 4.62-fold improvement over the noisy image. In addition, the average CNR values obtained using the UI of the salivary gland malignant tumor were 4.21, 9.00, 8.83, and 9.05 for the noisy image, TV, NLM, and the proposed adaptive NLM algorithms, respectively. Therefore, an excellent CNR value was derived from the UI of the malignant tumor using the proposed adaptive NLM algorithm, and this value was an approximately 2.15-fold improvement over the noisy image.

**Figure 7:**
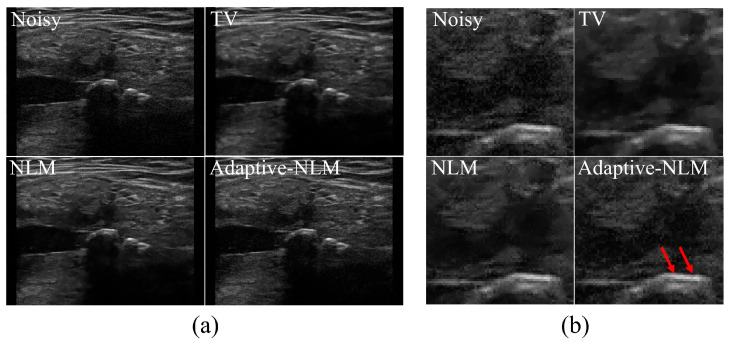
Results of applying noise reduction methods to the UIs of sialolithiasis occurring in the submandibular area of the salivary glands. (**a**) Noisy image, acquired using conventional TV, the NLM algorithm, and the proposed adaptive NLM algorithm, and (**b**) UI resulting from enlarging the ROI_A_ area in Figure 3c. When using the adaptive NLM algorithm in the red arrow area, a clear edge line is visible in the calcification area.

Figure 7 shows the UIs of a patient with sialolithiasis of the submandibular gland according to the noise reduction method. Figure 7a shows the UIs obtained by applying the noise reduction method, including the noisy image and the proposed adaptive NLM algorithm, and (b) shows the enlarged image obtained using the ROI_A_ area shown in Figure 3c. The enlarged area in Figure 7b is where the calcification is most clearly visible.

**Figure 8:**
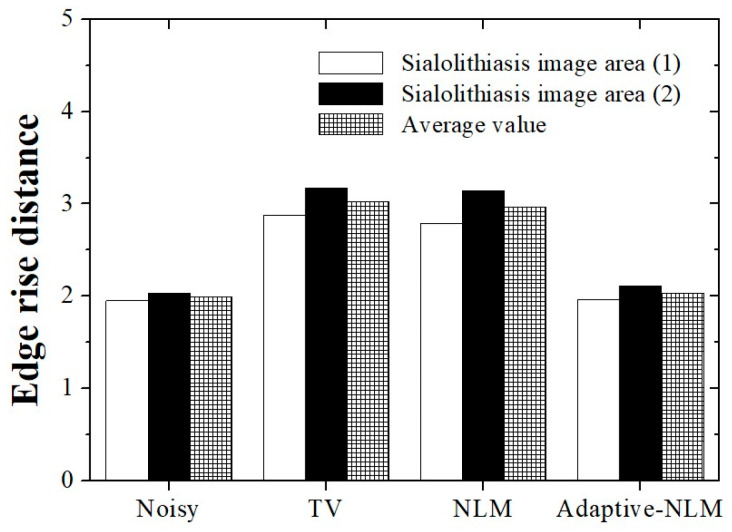
Graph showing edge rise distance (ERD) values calculated from the UI of the sialolithiasis in the salivary gland acquired using each noise reduction algorithm. An ERD value almost similar to that of the noisy image was derived from the UI using the adaptive NLM algorithm.

Figure 8 shows a graph of the ERD results according to the noise reduction method for the UI of sialolithiasis in the salivary gland. The ERD results were calculated as the average of the values measured in the Line_A_ and Line_B_ areas (Figure 3c). The average RED values obtained using the UI of the sialolithiasis in the salivary gland were 1.99, 3.03, 2.97, and 2.04 for the noisy image, TV, NLM, and the proposed adaptive NLM algorithms, respectively. An ERD value similar to that of the noisy image was derived when the proposed adaptive NLM algorithm was applied to the UIs. Compared with the conventional NLM algorithm, the adaptive NLM method improved the preservation of edge information by approximately 31.37%.

Sialolithiasis has a significant impact on the normal functions of the salivary glands and accounts for approximately 50% of the diseases occurring in the main salivary glands [36,37]. Symptoms of sialolithiasis have been reported at rates of 7.3 and 14.1 per 100,000 person-years. Because the severity of symptoms varies, they can be difficult to diagnose [36]. Patients with asymptomatic sialolithiasis can develop a more serious disease if surgery or treatment is not performed immediately; thus, ultrasound imaging technology with improved image quality is essential. In addition, in cases of sialolithiasis located behind the salivary gland duct, surgery is limited because of nerve damage and a limited surgical field of view. It is expected that these shortcomings can be overcome through efficient noise reduction and edge information preservation using the proposed adaptive NLM algorithm.

One limitation of this study was that it was limited to the ultrasound imaging of salivary gland diseases. Additional research should be conducted on various scanning methods and diseases where speckle noise is most commonly observed in UIs. Ultrasound-guided fine-needle aspiration cytology is widely used to treat salivary gland diseases [38]. Surgical resection is required if the possibility of tumor cell metastasis in a pleomorphic adenoma and a high false-negative rate are confirmed. Thus, to avoid redundant tests in the treatment of salivary gland diseases, additional research should be conducted on the effectiveness of improved ultrasound images using the algorithm proposed in this study.

In this study, the applicability of the adaptive NLM algorithm to salivary gland UIs was analyzed in terms of noise level and edge preservation. Although the data using the adaptive NLM algorithm showed superior characteristics compared to existing methods in the COV, CNR, and ERD evaluation results, additional research must be performed for clinical application. A tumor, or sialolithiasis, in the salivary glands is a disease that can be observed in various numbers in one area. For this reason, when a problem occurs in the image quality of UIs, it is very important clinically to check the exact number of lesions in the salivary glands. Thus, additional study should be conducted to determine whether the quantitative noise level and edge preservation ability values applied to salivary gland UIs using the adaptive NLM algorithm have a direct correlation with clinical evaluation in the future.

## 4. Conclusions

In this study, an adaptive NLM algorithm, which was modeled to efficiently optimize the conventional NLM algorithm and improve image quality, was applied to salivary gland UIs. The proposed adaptive NLM algorithm efficiently removed noise levels, confirming its applicability to the UIs of patients with salivary gland malignant tumors and sialolithiasis. In conclusion, the adaptive NLM method is expected to be of great help in diagnosing salivary gland diseases using ultrasound as it can simultaneously achieve high noise reduction efficiency and edge information preservation.

## Data Availability

Publicly available datasets were analyzed in this study. This data can be found here: [https://www.ultrasoundcases.info/cases/head-and-neck/salivary-glands] (accessed on 2 June 2024).
